# Teenager with a Primary Renal Lymphoma: A Case Report

**DOI:** 10.7759/cureus.7181

**Published:** 2020-03-04

**Authors:** Liliana Arriola-Montenegro, Cecilia D Palacios-Revilla, Maria Paula Cateriano-Alberdi, Renzo P Valdivia-Vega, Jose Arriola-Montenegro

**Affiliations:** 1 Internal Medicine, Universidad Peruana de Ciencias Aplicadas, Lima, PER; 2 Nephrology, Hospital Nacional Edgardo Rebagliati Martins, Lima, PER; 3 Internal Medicine, Sociedad Nacional de Capacitacion, Lima, PER

**Keywords:** lymphoma, b-cell, renal

## Abstract

We report a case of an 18-year-old male patient who was diagnosed with primary renal lymphoma (PRL) through biopsy findings and imaging studies. The patient presented with clinical manifestations of distal renal tubular acidosis including polyuria, polydipsia, lower limb weakness, involuntary weight loss, asthenia and dyspnea. No personal background or relevant medical history was reported. A kidney biopsy showed high grade immature B-cell lymphoproliferative process (Non-Hodgkin’s Lymphoma) with a Ki67 value greater than 90%. Complementary studies excluded primary lymphoid migration sites, which confirmed the diagnosis of PRL. The oncology unit initiated treatment with a combination of medications due to lack of protocols for the specific treatment. Besides the fact that this condition is rare, it also shows a unique symptoms presentation and non-typical findings in imaging methods. Also, it is important to underline the fact that the treatment is not yet specified for such type of cancer and different combinations are needed to control the disease.

## Introduction

Primary renal lymphoma (PRL) is a rare disease in our media; it is defined as a non-Hodgkin’s lymphoma (NHL) involving the kidney without presence of primarily extrarenal lymphatic disease. It is estimated that PRL accounts for 0.7% of extranodal lymphoma in North America [1]. Meanwhile in South America, the percentage of patients suffering from this disease has not been estimated. This condition has been doubted along the years due to the fact that the kidney lacks lymphoid tissue; nevertheless, there has been certain case reports around the world with different clinical manifestations that support its existence [2]. It has been said that lymphomagenesis could originate from hematogenous route from subcapsular lymphatics, or by direct extension from retroperitoneal cavity, sometimes associated with chronic inflammatory diseases that could result in these types of lymphoma [3].

The most common clinical manifestations of the disease include acute renal failure, lumbar pain and mass. Usually, a diagnosis is made in 60 to 70 years old patients [1]. Although there are not many cases reported around the world, the clinical presentations of PRL have varied in each report. Atypical presentations among the latest literature include findings of PRL in children with distal renal tubular acidosis including polyuria and polydipsia, lower limb weakness, weight loss in older patients and findings of proteinuria with nephrotic syndrome [1,4]. According to Stallone’s diagnostic criteria, there should be lymphomatous kidney infiltration, renal unilateral or bilateral non-obstructive enlargement, and no other location of lymphoma at the time of the diagnosis [2].

Nowadays, imaging studies are widely used as a way to discard a secondary lymphomatous tumor, but the confirmation of the PRL is made through histopathological findings. Generally, PRLs are diffuse large B cell lymphomas, but other histological types, such as follicular lymphoma, small lymphocytic or mucosa-associated lymphoid tissue (MALT) have been seen as well [2].

One of the chronic inflammatory diseases associated with the PLR is systemic erythematous lupus, which presents autoantibodies and as a consequence, entails a hyperactive immune system in which lymphocytes are rapidly growing and dividing. This chronic inflammation may give way to malignant cell transformation and to NHL occurrence [5].

## Case presentation

An 18-year-old male patient from Lima, Peru presented to the emergency room (ER) with episodes of polydipsia, polyuria and involuntary weight loss (approximately 18 kg over the past year); a progressive lower limbs weakness as well as dyspnea became associated. He was admitted due to incapacity to walk, mild left lumbar pain and severe asthenia. According to the physical examination, he was lucid, and orientated in time, person and space, with evidence of pale skin, oral mucous dryness and lower limbs weakness. Also, visceromegaly and pustular lesions with erythematous base along his back were found. The arterial blood gas analysis (ABG) taken at the emergency room showed metabolic acidosis with a normal anion gap and hypokalemia. The urine tests manifested alkaline urine, which indicated a distal renal tubular acidosis.

**Figure 1:**
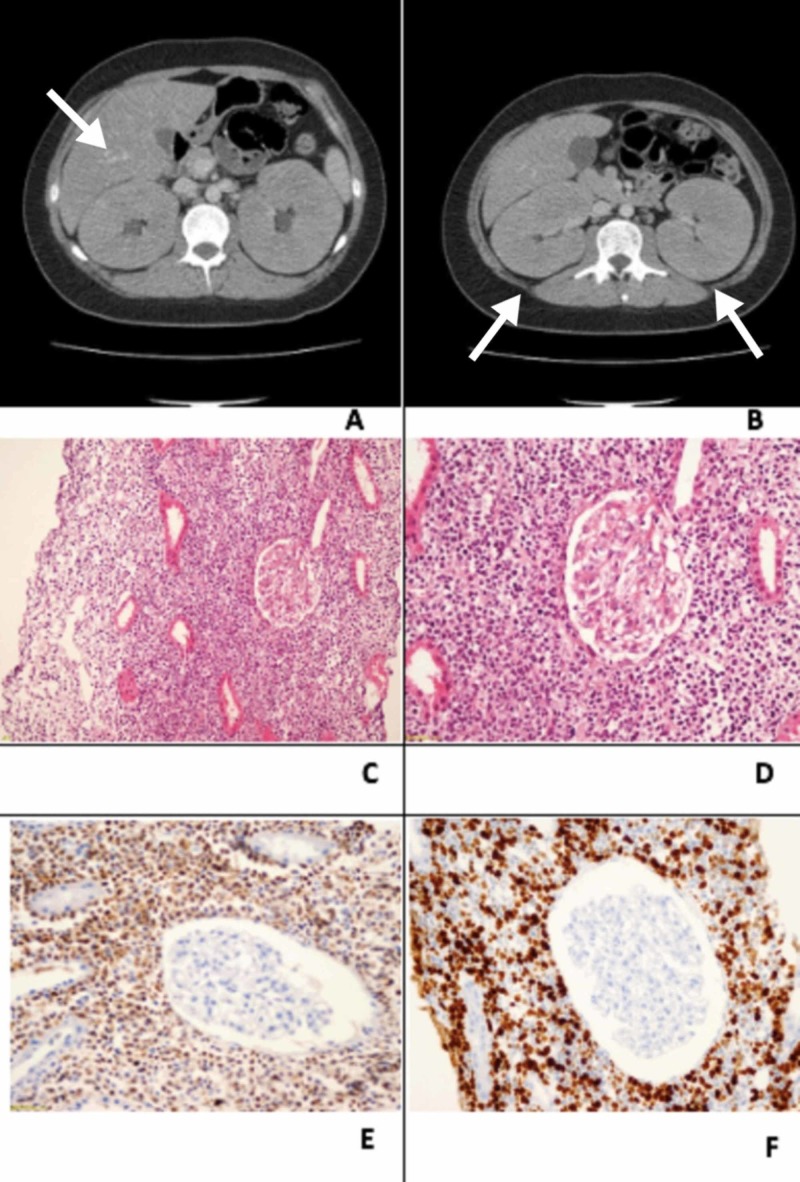
CT images and biopsy (A) CT images, arrow shows hepatomegaly. (B) CT images, arrows show nephromegaly. (C) 10x and (D) 20x hematoxylin and eosin staining shows diffuse infiltration of parenchymal tissue by lymphocytes. (E) and (F) 40x immunohistochemistry stained with a monoclonal antibody.

A week after admission, he was taken to the internal medicine service, where he went through a series of tests including laboratory exams, images and procedures. Systemic lupus erythematosus (SLE) was thought to be one of the first possible diagnosis with a lupus nephritis as a manifestation of the disease. Because of this, antibodies were asked and only antinuclear antibodies (ANA) came out positive. At the same time, different neoplastic and infectious diseases were ruled out with blood tests, tumor markers and other procedures. On the other hand, ultrasound images showed evidence of moderate hepatic steatosis and nephromegaly with signs of left renal cysts, which were ruled out during the CT scans, that demonstrated bilateral nephromegaly with hepatosplenomegaly and retroperitoneal, para-aortic and mesenteric lymphadenopathies that lacked significance (Figure 1). The renal ultrasound was repeated and a 9-mm lithiasis at a right renal calyx was found as well as a 5.6-mm stone in the left one.

Since there was not a definitive diagnosis, a renal biopsy was indicated (Figure 1). The results came out as a high-grade immature B-Cell lymphoproliferative process with a Ki67 value greater than 90%. With the possibility of a secondary renal lymphoma on mind, additional studies to evaluate the disease extension were requested (thoracic, abdomen and pelvic CT scans; cerebral CT scans and bone marrow aspiration). All of them came out negative and no primary lesion was found.

## Discussion

Renal lymphoma is a diagnosis made after completing a series of imaging exams, serums tests and a kidney biopsy. Even though a computer tomography is the best method for recognizing a pathological pattern, an ultrasound is usually the first image indicated by physicians and common findings have also been described [6]. A hypoechoic or anechoic mass associated with nephromegaly is the common image pattern in renal lymphoma; still, in this case report the ultrasound showed lithiasis not corresponding to the usual descriptions.

PRL tomographic findings in 66% of cases include isodense or hyperdense bilateral masses (CT without contrast), associated with irregular borders or encapsulated mass, and retroperitoneal lymphadenopathies. In 11-25% of patients, a retroperitoneal hypodense mass with parenchymal infiltration is seen [7]. In our case report, CT showed bilateral nephromegaly with hepatosplenomegaly and retroperitoneal, para-aortic and mesenteric lymphadenopathies that lacked significance. Furthermore, it is important to rule out the possibility of metastasis or primary lesions. In our case, several laboratory exams, imaging studies and tumor markers came out negative for primary or metastatic lesions.

The next decision taken was a renal biopsy, which is the most cost-effective method diagnosis for deciding patient’s management [8]. This step was important in our case to make the correct diagnosis about the histology of the malignancy. The biopsy showed findings of a non-Hodgkin lymphoma in the kidney. Since this type of malignancy is usually associated to primary autoimmune or infectious diseases, this possibility had to be ruled out before stating it was a primary renal lymphoma.

Autoimmune diseases are included in the chronic pathologies associated with the progression of secondary lymphoma. Systemic lupus erythematosus (SLE) and Sjogren syndrome (SS) were ruled out as only ANA came out positive, and B3 microglobulinuria and electrophoretic proteinogram were normal, but it is important to underline that these pathologies have shown strong evidence. According to different reviews, SLE, SS, rheumatoid arthritis and psoriasis are the most associated [9].

The average age of diagnosis of NHL associated with SS is 54 years [10]. Histologic type of NHL associated with SS that appears the most is the marginal B cell type [11,12]. Also, independent risk factors for developing NHL from SS include, salivary glands swelling, lymphadenopathies, Raynaud phenomenon, positivism for anti-Ro/anti-LA and rheumatoid factor, presence of monoclonal gammopathies and low values of C4 [13]. Other authors add palpable purpura, skin compromise, cryoglobulinemia and urine and blood monoclonal components [14]. These factors contribute to the formation and activation of immunocomplexes, which triggers hypocomplementemia and B-cell proliferation [13].

Primary infectious diseases were tested and IgG for Epstein-Barr virus (EBV) came out positive (1:320). It is well known that EBV can give origin to a secondary lymphoma. The pathology mechanism is explained by the invasion of B cell by the virus, affecting the immune system and originating the lymphoma [15,16]. Nevertheless, there is no significant association between IgG values and NHL development, because laboratory values vary among countries and because only IgG anti-VCA is related with NHL [17].

As we mentioned in the case presentation, the patient had a distal renal tubular acidosis (dRTA) confirmed by a metabolic acidosis found in the ABG, as well as a normal anion gap, hypokalemia and alkaline urine among other clinical findings that will be mentioned below. A dRTA is, as its name says, a malfunction of the kidney’s distal tube that causes metabolic acidosis, specifically an impaired secretion of hydrogen ions that takes place because of a defect in the ATPase pump [18,19]. This type of acidosis could be either hereditary or acquired and plenty of causes have been reported [20].

The reasons and mechanisms of the existence of a dRTA in the pathologies mentioned are uncertain despite the various hypothesis made around the subject [20]. Even though SLE is one of the most common etiologies of distal renal tubular acidosis, the patient came out positive only for ANA. Clinical manifestations of dRTA, besides the ones already mentioned above, are polydipsia, polyuria and it is not uncommon presentation of nephrolithiasis, which the patient also had [2]. All of these characteristics were present, as they seem to be the main symptoms. After the biopsy results it was inquired that, since it was a case of primary renal lymphoma, the lymphoproliferative process could have been the reason of the damaged distal tube of both kidneys resulting in a dRTA in this patient and not because of a primary event such as SLE.

## Conclusions

Finally, after ruling out all the possible primary causes, they made the diagnosis of a primary renal lymphoma with clinical manifestations of dRTA and particular CT descriptions. The oncology department took the case to start the patient on chemotherapy following a non-Hodgkin lymphoma protocol. The importance of this report lies on the uncommon presentation and age of onset, as well as it being the first case report of this kind of lymphoma in Peru.
